# Preparation and characterization of graphene oxide-based cation, chelating, and anion exchangers for salt removal

**DOI:** 10.1016/j.heliyon.2025.e42070

**Published:** 2025-01-17

**Authors:** Mahmoud A. Al Azwani, Saleh Al Busafi, El-Said I. El-Shafey

**Affiliations:** Department of Chemistry, College of Science, Sultan Qaboos University, PO. Box 36, PC123, Oman

**Keywords:** Ion exchange, Sorption, Removal, Graphene oxide, Chelation

## Abstract

Due to the shortage of freshwater resources, and the limitations of commonly used ion exchangers, this study aims to prepare, characterize, and test new graphene-based ion exchangers for salt removal. Graphene oxide (GO) was prepared using an oxidative exfoliation method. Using amide coupling, the GO surface was functionalized with taurine to produce a cation exchanger (GOT), and pentaethylenehexamine (PEHA) to produce a chelating ion exchanger (GOP) which was converted to a quaternary ammonium salt (GOQ) which acts as an anion exchanger. The surface area of GO was 220 m^2^/g, however, decreased tremendously on surface functionalization. X-ray diffraction (XRD) showed that the produced ion exchangers possess amorphous nature. Energy dispersive spectroscopy (EDS) showed the presence of nitrogen in GOP, GOQ, and GOT; and sulfur on GOT. X-ray photon spectroscopy (XPS) showed the presence of -SO_3_ and S-O on GOT, amine on GOP, and quaternary ammonium group (-NHR_2_^+^) on GOQ. TGA shows that GO functionalization is covalent. The produced ion exchangers show an efficient removal of both the cations and anions from the individual salt solution of Ca(NO_3_)_2_, MgSO_4_, and NaCl. GOP sorbs both Ca^2+^ and Mg^2+^ via chelation while their anions are sorbed via ion pairing. GOT sorbs the cations via ion exchange while anions are sorbed via ion pairing. GOQ sorbs the anions via electrostatic interaction while the cations are sorbed via ion pairing. Under the experimental conditions in this study, GOP shows the best removal of Ca^2+^ (80.2 %) and Mg^2+^ (64 %) while GOT shows the best removal of Na^+^ (30 %). For anions, GOQ shows the best removal of NO_3_^−^ (53.5 %), SO_4_^2−^ (84 %), and Cl^−^ (81.8 %). The GO-based ion exchangers seem promising for salt removal from water in addition to being robust at high temperatures and pH.

## Highlights

• GO was functionalized to produce ion exchangers GOT, GOP and GOQ.
• GOT, GOP and GOQ are cation, chelating and anion exchangers.
• Characterization proved successful functionalization.
• GOP sorbs Mg^2+^ and Ca^2+^ via chelation while GOT sorbs Na^+^ via ion exchange.
• GOQ sorbs Cl^−^, SO_4_^2−^ and NO_3_^−^ via ion exchange while metals via ion pairing.

## 1 Introduction

Modern industrial development and the increase in the world's population caused increased emissions that led to water pollution, climate change, global warming, and seawater intrusion. As a result, freshwater shortage has become inevitable worldwide [1] with almost 40 % of the world's population affected by 2030 [2]. The access to clean water is critical for our life. Every person requires from 20 to 50 L of clean water per day covering many purposes such as drinking, bathing, cooking, and personal hygiene. It is also critical for many industries such as food production, agriculture, power generation, medical uses, firefighting, and recreation purposes [3,4]. Clean water from infectious agents and toxic pollutants protects human and animal lives. Industrial pollution is considered the main threat to freshwater supply and contamination [5]. Due to the over-pumping of groundwater to meet the agricultural demand, and the sea rise because of climate change, seawater intrusion has adversely affected the groundwater quality turning it brackish or even saline [6]. Irrigation with brackish water for a relatively long time not only damages crop performance but also causes salt accumulation in soil, and consequently, the deterioration of soil productivity [7]. Salinity affects the vegetation at all stages of plant growth including germination, seeding, vegetative and mature stages because of the osmotic effects, ion toxicity, and nutrition discrepancy [8]. Considering that freshwater accounts only for 3 % of available water resources on Earth, various methods of desalination and water treatment are implemented as reliable and sustainable freshwater resources [9]. Desalination methods can be categorized as thermal and membrane desalination techniques. Thermal techniques involve heating saline water and collecting condensed water such as vapor compression, multi-stage flash distillation, humidification-dehumidification process, and multi-effect distillation. These methods consume energy and generate hot brine which affects the marine environment on disposal [9]. Membrane desalination methods involve the use of electrical power and membranes such as nanofiltration, reverse osmosis, electrodialysis, and forward osmosis [9,10]. Membrane processes, even though mostly used, suffer from scaling and membrane clogging in addition to brine generation which can also affect the marine environment [9,11]. Ion exchange as a technique for brackish water desalination is preferred due to its high efficiency, low cost, less volume of sludge volume, and its reuse efficiency [12]. However, it is nonselective, highly sensitive to the pH of the solution, and is limited mostly to low salinity levels [13]. Ion exchangers can be cationic, anionic, and chelating. Cationic exchangers can be strongly acidic resins that possess sulfonic acid groups (−SO_3H_), or weakly acidic resins with carboxylic acid groups (–COOH) [14]. Anion exchangers can contain protonated nitrogen atoms which can be a weak (−NH_3_^+^) or strong (−N(CH_3_)_3_^+^) anion exchanger. Chelating ion exchanger possesses an immobilized ligand which can remove metals by chelation or complex formation such as aminophosphonate chelating resin [15] and covalently immobilized EDTA onto GO [12,16]. Ethylenediamine was also immobilized onto a dehydrated carbon surface which showed an efficient removal of heavy metals from aqueous solutions [[17], [18], [19]]. Due to its excellent chemical stability, high tensile strength, and multi-functional surface, GO has drawn significant attention in water desalination and purification [20]. Because of being loaded with oxygen-rich functional groups such as hydroxyl and epoxy groups, GO displays characteristics comparable to graphene [21]. GO covalent ion exchangers overcome the limitation experienced by the common ion exchangers such as the polymeric ion exchange resin which can suffer damage at temperatures higher than 80 °C [22], and silica-based ion exchangers which can be damaged at alkaline pH [23]. GO covalently bonded ion exchangers are robust and stable with variations in both temperature and pH [12]. The incorporated oxygen on the structure of GO sheets as surface functional groups (such as carbonyl and epoxide groups on the core, and the hydroxyl and carboxyl groups on the edges) acts as active centers for GO functionalization [20]. Several studies showed that different types of aliphatic diamines with different lengths can be immobilized onto GO nanosheets to improve their flexibility and hydrophilicity [12,16,[24], [25], [26], [27]]. GO was functionalized using chitosan for salt removal from water [28,29]. In a previous study, GO was functionalized using EDTA and chitosan showed a good performance for the removal of Pb^2+^ and Cu^2+^ [30]. In another study, Zeolitic imidazolate frameworks were anchored onto amine functionalized GO as a hybrid nanocomposite which was successfully used as a catalyst for the degradation of Rhodamine B dye and p-Nitrophenol [31].

In this study, GO was prepared from graphite flakes using the PAOM method (performed acidic oxidizing medium) which is a modified Hummers' method [32] as the original Hummers' method is dangerous and requires ice bath application to minimize its risks [33]. Using amide coupling processes, GO was functionalized with taurine (2-aminoethanesulfonic acid) to prepare a strong cation exchanger, and pentaethylenehexamine (PEHA) to prepare a chelating ion exchanger. The covalently immobilized PEHA was converted to quaternary ammonium salt as an anion exchanger. The produced ion exchangers are surface characterized and tested for desalting Ca(NO_3_)_2_, MgSO_4,_ and NaCl from synthetic solution.

## 2 Materials and methods

### 2.1 Chemicals

All chemicals used that were purchased from Sigma-Aldrich, are of analytical grade. Materials used include graphite, H_2_SO_4_, P_2_O_5_, KMnO_4_, NaNO_3_, H_2_O_2_, HCl, NaOH, SOCl_2_, 2-aminosulfonic acid, pentaethylene hexamine, pyridine, toluene, CH_3_I, K_2_CO_3_, NaOH and isopropyl alcohol. Salts used include Ca(NO_3_)_2_, NaCl and MgSO_4_.7H_2_O. Stock individual salt solutions of each of NaCl (Na^+^ 500 mg/L, Cl^−^ 771 mg/L), MgSO_4_ (Mg^2+^ 500 mg/L, SO_4_^2−^ 1975 mg/L), and Ca(NO_3_)_2_ (Ca^2+^ 500 mg/L, NO_3_^−^ 1546 mg/L) were prepared in ultrapure water. Other concentrations were prepared by suitable dilution in ultrapure water.

### 2.2 Preparation of graphene oxide

Graphene oxide (GO) was prepared using the preformed acidic oxidizing medium (PAOM) method which is a modified Hummers’ method [12,32]. An oxidizing mixture of concentrated H_2_SO_4_ (138 mL, 98 %), 6 g of P_2_O_5,_ and 18 g of KMnO_4_ were mixed and kept for 3 min. A homogeneous mixture of 6 g of graphite (G) and 3 g of NaNO_3_ was added carefully to the oxidizing mixture and the mixture was kept for 10 min. The reaction mixture was heated in an oil bath at 35 °C for 1 h under continuous stirring. 300 mL of deionized water was added slowly and carefully to the reaction mixture to prevent overheating, after which, the mixture was then heated in an oil bath at 85 °C for 15 min. After cooling at room temperature, 120 mL of H_2_O_2_ (10 mL, 35 %) was added slowly. Produced GO was filtered under vacuum and washed with 200 mL (25 % HCl). GO was washed further with deionized water and the supernatant was decanted several times until the pH was neutral. GO was sonicated for 10 min in deionized water, after which, it was filtered, dried at 120 °C for 24 h, transferred to a desiccator to cool, and finally stored in a dry, clean, and well-closed polyethylene jar.

### 2.3 GO functionalization

Taurine (2-aminosulfonic acid) was immobilized on GO surface to produce a strong cation exchanger (GOT). Taurine solution was prepared by dissolving 10 g of taurine in 110 mL of 0.1 NaOH under stirring while 20 mL of pyridine was added to the solution. 8 g of GO was mixed with 50 % SOCl_2_ in toluene (50 mL) under reflux in a 250 mL round bottom flask for 2 h at 70 °C. The product (GO-COCl) was allowed to cool and the solvent was dried using a rotary evaporator at 40 °C. After solvent drying, taurine solution was immediately added to the product and was kept under reflux with stirring at 90 °C for 2 h. The product was allowed to cool down. During this process, taurine was immobilized onto the GO surface via amide coupling and GOT was produced. GOT was washed with acetone to remove residual pyridine, followed by 100 mL of 0.1M HCl to release un-immobilized taurine. Finally, GOT was washed thoroughly with hot ultrapure water until no acidity was detected in the wash water (using Blue litmus paper). GOT was filtered and dried at 105 °C, after which, it was kept to cool in a desiccator, and finally stored in a dry, clean, and well-closed polyethylene jar.

GO was also functionalized using pentaethylenehexamine (PEHA) via amide coupling to produce a chelating ion exchanger (GOP) which was converted to its quaternary ammonium salt (GOQ) as follows. 8 g of GO was mixed with 50 mL of 50 % SOCl_2_ (in toluene) in a round bottom flask and was kept under reflux for 2 h at 70 °C. The product was allowed to cool and the solvent was dried using a rotary evaporator at 40 °C. GO-COCl was immediately mixed with 100 mL of 10 % pentaethylenehexamaine (PEHA) in toluene and was refluxed for 2 h at 90 °C. The product was allowed to cool down at room temperature and the solvent was decanted followed by drying using a rotary evaporator at 40 °C. During this stage, PEHA was immobilized covalently onto GO surface producing GOP. GOP was washed with 50 mL acetone to remove residual reagents, followed by 100 mL 0.1 M HCl to remove the free unfunctionalized PEHA molecules. GOP was further washed with 100 mL 0.1 M NaOH to deprotonate the immobilized amine groups onto GO. Finally, GOP was washed with hot deionized water several times until no basicity was detected in the wash water (using litmus paper). GOP was eventually filtered and dried at 105 °C, after which it was transferred to a desiccator to cool, and then stored in a dry, clean, and well-closed polyethylene jar.

**Fig. 1:**
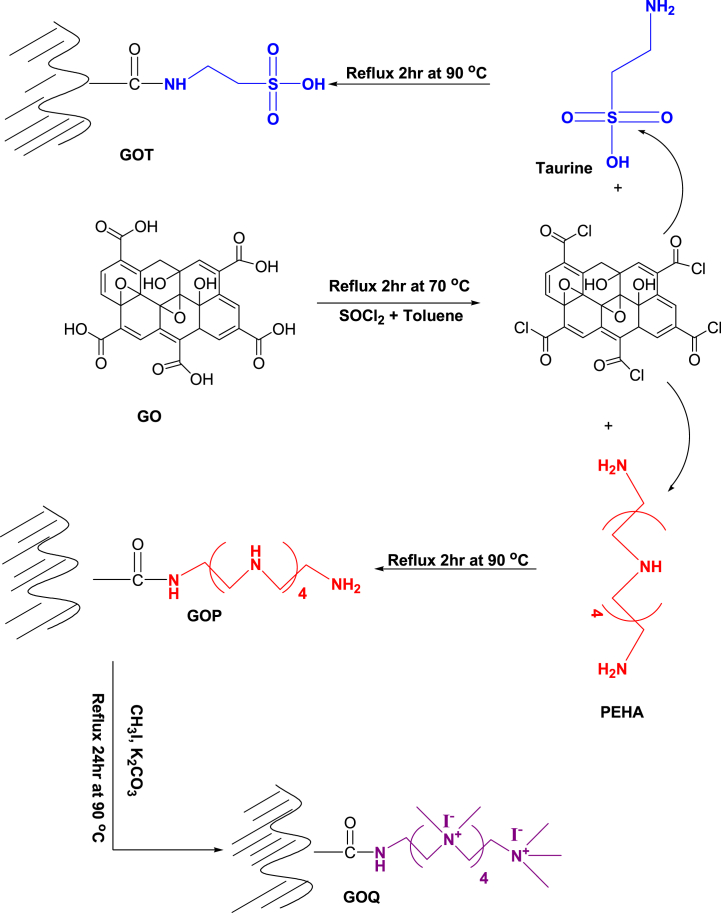
Schematic representation of GO-based ion exchangers.

GOP was converted to GOQ as follows. GOP (∼10 g) was mixed with 30 g of CH_3_I, 30 g of K_2_CO_3,_ and 100 mL isopropyl alcohol in a 250 mL round bottom flask. The mixture was kept at 90 °C under reflux with constant stirring for 24 h. By the end of this step, the amine groups on GOP were converted to quaternary ammonium iodide salt designated as GOQ. Produced GOQ was allowed to cool at room temperature followed by washing with isopropyl alcohol and then by ultrapure water to wash away unreacted reagents. Finally, the collected GOQ was dried in an oven at 105 °C. Dry GOQ was transferred to a desiccator to cool and then stored in a dry, clean, and well-closed polyethylene jar. The scheme of preparation of the ion exchangers is presented in Fig. 1.

### 2.4 Physicochemical characterization

The surface area and porosity were investigated using an ASAP 2020 instrument (Micrometrics, USA) via nitrogen adsorption at 77 K. Degassing stage was performed for G and GO at 200 °C for 8 h under vacuum, while for GOT, GOP, and GOQ, it was carried out at 90 °C for 24 h under vacuum (0.1 atm) to avoid possible changes at a higher degassing temperature [34]. Field emission-scanning electron microscopy (FE-SEM) and energy dispersive spectroscopy (EDS) were carried out using JEOL/EO JSM 5600 scanning electron microscope (Jeol, Japan) at an accelerating voltage of 20 kV and a JEOL/O JSM 5600 editor energy disperse analysis system (Joel, Japan) attached to the scanning microscope. X-ray diffraction was conducted using a Philips PW 1830 generator with a Philips PW 1050 powder goniometer (Philips, USA) with copper *Kα* as the incident radiation. The materials were also tested for lattice and morphology using a transmission electron microscope, TEM (JEM 1400, Joel, Japan). XPS analysis for the ion exchangers was also carried out using a multi-probe X-ray photoelectron spectroscope (XPS, Omicron Nanotechnology, Germany) with (Al) *Kα* (hν = 1486.6 eV) radiated at 15 kV. XPS results were analyzed using CasaXPS software (Casa Software Ltd, UK) with a Shirley-type background. The calibration of the binding energy of the spectra was performed with the C1s peak at 284.6 eV. Fourier transform infrared (FTIR) of dried samples was conducted using an FT-IR spectrometer (PerkinElmer, Spectrum One, USA) between 500 and 4000 cm^−1^ with background subtraction. The surface zero point of charge (pH_zpc_) was investigated following the procedure of Moreno-Castilla et al. [35]. Apparent density, cation exchange capacity (CEC), and Boehm titrations were carried out following standard methods [[36], [37], [38]]. Anion exchange capacity (AEC) was determined using the chloride adsorption-desorption method [39]. Surface basicity was also determined. 0.1 g of carbon was added to 25 mL HCl (0.1 M) in 30 mL polyethylene vials and the mixture was left for 48 h under agitation at 25 °C in a shaking water bath (100 rpm). Finally, aliquots of residual acid were analyzed by titration against 0.1M NaOH using methyl orange indicator. Thermogravimetric analysis (TGA) was carried out using SDT Q600 Simultaneous DSC-TGA equipment (TA instruments, USA). Heating was carried out under a nitrogen atmosphere with a flow rate of 100 mL/min from room temperature to 800 °C at a heating rate of 20 °C/min.

### 2.5 Salt sorption onto ion exchangers

Different masses (0.3–0.9 g) of sorbents were mixed with 25 mL individual solutions of NaCl (Na^+^ 50 mg/L, Cl^−^ 77.1 mg/L), MgSO_4_ (Mg^2+^ 50 mg/L, SO_4_^2−^ 197.5 mg/L), and Ca(NO_3_)_2_ (Ca^2+^ 50 mg/L, NO_3_^−^ 154.6 mg/L) at room temperature. The sorption mixtures were kept under mechanical agitation (100 rpm) for 2 h to reach equilibrium. After equilibrium, supernatants were separated for analysis. Metals were analyzed using Agilent 5800 inductively coupled plasma-optical emission spectrometer (ICP-OES), while the anions were analyzed using ion chromatography (Dionex Corporation, USA). All the experiments were carried out at least twice.

## 3 Results and discussions

### 3.1 Sorbent characterization

The oxidative exfoliation of graphite produced single-layered GO which possesses functional groups such as hydroxyl (–OH), carboxyl (COOH) on the edges, and carbonyl (C=O) and epoxy (C-O-C) on the core of GO sheets [20]. The covalent immobilization of taurine and PEHA to produce GOT and GOP, respectively, occurs mostly on the edges of GO sheets.

### 3.2 Surface area and porosity

The surface area (S_BET_) and other pore widths are presented in Table 1 and the nitrogen adsorption-desorption isotherms are presented in Appendix A1. Graphite (G) exhibits a very low surface area (0.110 m^2^/g) which agrees with literature values [12,40,41] because of the compacted graphene layers in the graphitic structure. On the other hand, GO exhibits a larger surface area of 220.9 m^2^/g which is typical for GO [12]. The single-layered GO allows more uptake of nitrogen and, consequently, high surface area. However, after functionalization, GOT, GOP, and GOQ show extremely low surface areas due to their hydrophilic surface nature which interacts weakly with the non-polar nitrogen molecules [19]. In a previous study, the surface area of GO has tremendously decreased after EDTA covalent immobilization [12]. In another study, the surface area of activated carbon (823 m^2^/g) also decreased as a result of surface functionalization using ethylamine (9.89 m^2^/g) and ethylene diamine (4.02 m^2^/g) [34]. The tremendous decrease in surface area of the GO-based ion exchangers is not only related to surface hydrophilicity [12] but also, to the restriction of nitrogen access to the active sites caused by the immobilized compounds [34]. While G exhibits microporous characteristics, GO, GOT, GOP, and GOQ show mesoporous features. Similar results were found for functionalized GO with ethylene diamine and EDTA [12]. The apparent density of graphite (0.66 g/cm^3^) has decreased on oxidative exfoliation to 0.28 g/cm^3^ and increased again after surface functionalization (Table 1). The average particle size (L_D_) in micrometers (μm) was calculated from Equation (1) [42,43].

(Eq. 1) $$L_{D}=6/\left(S_{\text{BET}}\;.\;\rho \right)$$

where S_BET_ is the specific surface area (m^2^/g) measured using the BET method, and ρ is the particle density (g/cm^3^).

**Table 1:** Surface texture of graphite and its derivatives.

Sorbent	BET Surface area∗ (m^2^/g)	Average pore width (A^o^)	Apparent Density (g/cm^3^)	Average particle size (μm)
G	0.110	12.93	0.66	82.64
GO	220.9	149.9	0.28	0.097
GOT	0.220	73. 8	0.55	49.59
GOP	0.234	113.4	0.49	52.33
GOQ	0.200	487.6	0.31	96.78

Surface area was measured via nitrogen adsorption at 77 K.

Graphite particle size is large (82.99 μm), nevertheless, GO shows a smaller particle size of 0.027 μm indicating a successful exfoliation process. The particle size has again increased on GO functionalization for GOT (49.6 μm) and GOP (52.33 μm). The conversion of GOP to GOQ has led to a further increase in the particle size (96.8 μm) indicating a successful formation of quaternary ammonium salt.

### 3.3 TEM, SEM, EDS, and X-ray diffraction

**Fig. 2:**
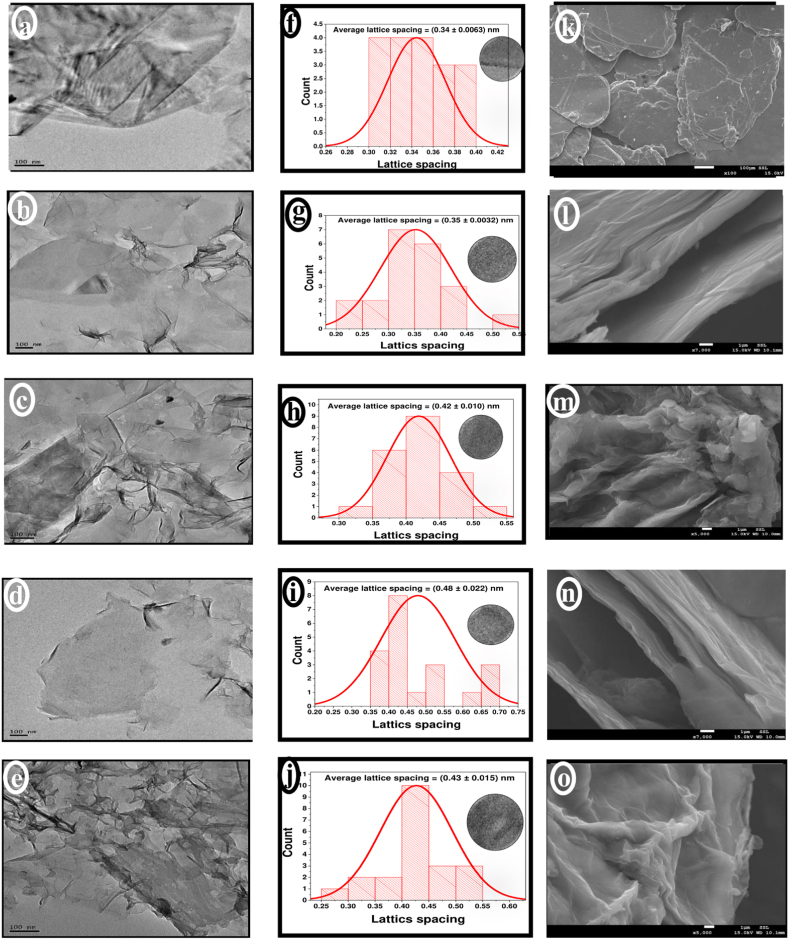
(a–e) TEM image, (f–j) the lattice spacing distribution, and (k–o) SEM micrographs of G, GO, GOT, GOP and GOQ, respectively.

The morphology of G, GO, and GO-based ion exchangers was examined by TEM as presented in Fig. 2(a–e). Displayed in Fig. 2a, the graphite image shows dark-colored regions indicating a graphite pile of several layers [44]. After the oxidative exfoliation, the dark regions of graphite have decreased with the presence of wrinkling indicating a successful exfoliation process (Fig. 2b) [45]. As surface functionalization took place mostly at the edges of GO, a dark color appears on the edges of GOT, GOP, and GOQ particles due to the accumulation of the immobilized taurine, PEHA, and the quaternary ammonium salts at the edges, respectively (Fig. 2c-e).

As presented in Fig. 2(f–j), the average lattice spacing, measured using TEM images, has increased from 0.34 ± 0.006 nm for G to 0.35 ± 0.003 nm for GO. The presence of oxygen functional groups on the GO (especially on the edges) such as –COOH and -OH which are sp^3^ formations led to an increase in the inter-layer spacing [46]. However, after surface functionalization, the average spacing has further increased to 0.42 ± 0.01 nm, 0.48 ± 0.02, and 0.43 ± 0.02 nm for GOT, GOP, and GOQ, respectively. The immobilized molecules on GO pushed the graphene layers further away from each other turning the structure to be mostly amorphous. For GOP, the lattice spacing showed an increase to 0.65–0.70 nm between graphite sheets (Fig. 2i) and this is probably related to the presence of an extent of the immobilized PEHA molecules between the sheets.

SEM reveals the external surface information of G, GO, and functionalized GO materials (Fig. 2k–o). However, SEM analysis varies depending on selected sections from the surface, especially for non-homogeneous surfaces. As clearly shown in Fig. 2(k–o), G shows an ordered and compacted layered structure [12,47], however, GO shows wrinkled layered structures indicating a successful oxidative exfoliation process [12,47]. More wrinkling appears for GOT, GOP, and GOQ as a result of surface functionalization.

EDS spectra of GO, GOT, and GOP were represented in appendix (A2) as examples. Graphite shows 100 % of carbon (Table 2) while for GO, carbon dropped to 61.8 % with a content of oxygen 34.3 % as a result of oxidation. Compared with GO, GOT shows an increase in carbon content to 67.8 % with a decrease in oxygen content to 29.4 %, however with a 1.7 % sulfur indicating the immobilization of taurine. For GOP, carbon shows a very clear decrease to 41.43 %, and oxygen to 32.1 %, however, with nitrogen content of 18.1 % as a result of PEHA immobilization. Compared with GOP, GOQ shows an increased carbon content of 52.3 %, decreased oxygen of 29.7 %, and nitrogen to 13.7 % (Table 2). EDS technique analyzes about 2 μm depth of sorbent surface [48], however with some limitations. It is difficult to detect light elements with this technique in addition to interference from overlapping peaks of other elements. The emitted X-rays from the specimen atoms are emitted usually in all directions and some of these X-rays may not escape the sample and cannot be detected. Therefore, minor elements might not be detected while major elements in the specimen can be overestimated [49].

**Table 2:** EDS elemental analysis.

Element	Weight (%)				
	G	GO	GOP	GOQ	GOT
C	100.0	61.8	41.3	52.3	67.8
O	NA	34.3	32.1	29.7	29.4
N	NA	NA	18.1	13.7	NA
S	NA	0.9	NA	NA	1.7
Cl	NA	0.1	NA	NA	0.2
Mg	NA	1.4	NA	NA	NA
Al	NA	0.7	NA	NA	0.4
Na	NA	0.5	0.2	NA	0.3
K	NA	NA	NA	4.3	NA
Cr	NA	NA	8.2	NA	NA

NA: Not Available.

**Fig. 3:**
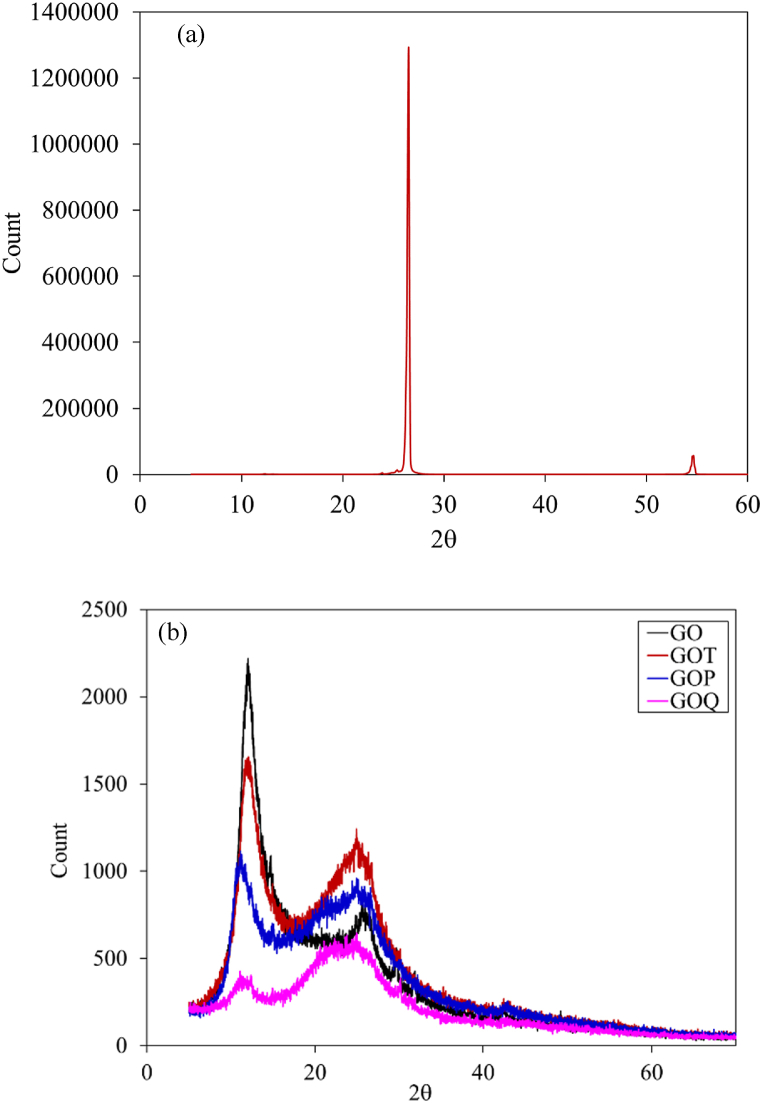
X-ray Powder diffraction of (a) G and (b) GO, GOT, GOP, and GOQ.

XRD spectra are presented in Fig. 3. G shows a strong and well-defined peak at 2θ of 26.5° [49] indicating a well-arranged layer structure with 0.336 nm d-spacing, with 002 orientation [12,48], Fig. 3a. GO shows an obvious peak shift from 2θ of 26.5^o^ to 12.29^o^, with increased interlayer spacing between the GO layers (0.72 nm), because of the insertion of oxygen groups such as carboxylic groups, carbonyl, epoxide, and hydroxyl [12,50], Fig. 3b. For GOT, the peak intensity at 2θ of 12.3^o^ has decreased, and another wide peak appeared at 24.9^o^, Fig. 3b. For GOP, the peak shifted from 2θ of 12.3^o^ to 11.3^o^ with a decreased intensity and a wider peak appeared at 25.1^o^. Finally, for GOQ, the peak at 2θ of 12.3^o^ for GO has diminished with a shift to 2θ of 11.07^o^ with a small and wide peak appearing at 2θ of 24.9^o^, Fig. 3b. The wide peaks indicate the presence of amorphous structure of GOT, GOP, and GOQ [12] with a further increase in interlayer spacing to 0.78, 0.84, and 0.81 nm, respectively.

### 3.4 FTIR

**Fig. 4:**
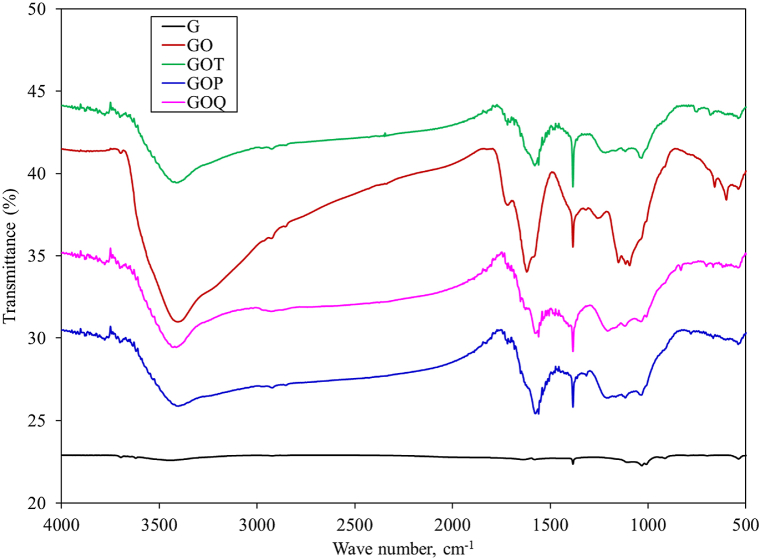
FTIR spectra of G, GO, and GO-based ion exchangers.

FTIR spectra in Fig. 4 illustrate the absence of significant bands on G and conclude that G is effectively oxidized to GO [50]. GO displays an intense band at about 3400 cm^−1^ (O-H stretching vibration), while for GOT, GOP, and GOQ, the band is related to O-H and N-H stretching vibrations [34]. The small bands at 2920 and 2850 cm^−1^ are related to C-H stretching vibration. The band at 1723 cm^−1^ for GO which is related to C=O stretching vibration in –COOH became less intense for GOT, GOP, and GOT indicating a successful functionalization [34]. The band at 1620 cm^−1^ for GO is related to C=C or C=O stretching vibration [49]. This band has shifted to 1560 cm^−1^ for GOT, GOP, and GOQ. The band at 1385 cm^−1^ which appears for GO, GOT, GOP, and GOQ but as a shoulder for G, is related to C-H bending vibration [12]. For GO, the bands at 2252 cm^−1^ and those between 1115 and 1000 cm^−1^ correspond to C-O bonds [51]. GOP also shows another band at 1316 and 1213 cm^−1^ which are related to C-N stretching vibration. The band between 1210 and 1220 cm^−1^ for GOT and GOQ is related to C-N stretching vibration. The bands between 1200 and 1000 cm^−1^ are related to C-O vibrations [51]. For GOT, the bands at 1168, 117, and 1035 cm^−1^ are related to S=O stretching vibration [51], while the band at 680 is related to S-OH vibration [51].

### 3.5 XPS analysis

**Fig. 5:**
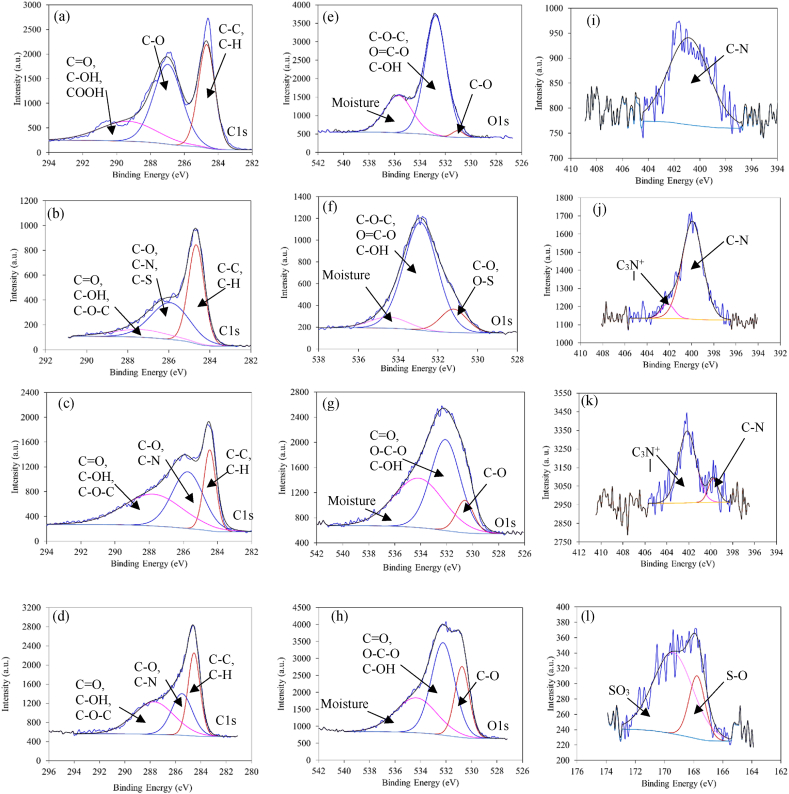
XPS results of C 1s (a–d), O 1s (e–h) for GO, GOT, GOP, and GOQ respectively, N 1s (i–k) for GOT, GOP, and GOQ, respectively, and (l) S 2p for GOT.

XPS analysis reveals the surface properties at a depth of <10 nm [12]. The surface elemental analysis of GO, GOP, GOQ, and GOT is presented in Table 3. Nitrogen was detected in GOP, GOQ, and GOT but undetected in GO while sulfur was detected only in GOT. The high-resolution XPS spectra are presented in Fig. 5. The peaks detected at the binding energies of about 284.6, 532, 400, and 169 eV are related to C 1s, O 1s, N 1S, and S 2p, respectively. The deconvolution of the C 1s peak shows three individual component peaks (Fig. 5a–d). The peaks in the range of 284.5–284.7 eV correspond to sp^3^ C-C and/or C-H [52] in GO and its derivatives. The peaks in the range of 285.5–285.7 eV correspond to C-O, C-N, or C-S, as for GOT [53,54]. However, the peak in the range of 287.0–287.8 eV corresponds to C=O, C-OH and C-O-C [55]. For GO, the peak at 289.3 eV is related to COOH group [56], such a peak was not found for GOT, GOP, or GOQ. The deconvolution of O 1s XPS spectra shows three distinguished peaks as shown in Fig. 5(e–h). C-O peak appears at 531.1, 530.6, 530.8, and 531.2 eV for GO (Fig. 5e), GOT (Fig. 5f), GOP (Fig. 5g), and GOQ (Fig. 5i), respectively [52,53]. The peak of GOT at 531.2 eV can be also correlated to O-S in sulfite structure [57]. The peaks at 532.8, and 532.9 eV for GO, and GOT, respectively, can be attributed to COO- (unfunctionalized extent –COOH on GOT), C-OH, and C=O [58], while the peaks at 532.1, and 532.2 eV can be attributed to C-OH, C-O-C, and C=O [53], on GOP, and GOQ, respectively [53]. The peaks at 535.7, 534.5, 534.2, and 534.4 eV for GO, GOT, GOP, and GOQ, respectively, are related to physisorbed water [59,60]. The deconvolution of N 1s of GOT (Fig. 5i) shows a single peak appearing at 400.6 which is related to the amide group [61]. However, the deconvolution of N 1s peak for GOP and GOQ, presented in Fig. 5j & k, respectively, shows two peaks. The peak at 399.8–399.9 eV corresponds to primary and secondary amine while that around 402.1 eV represents mainly quaternary positively charged nitrogen [62]. The area of amine on GOP accounts for 91.4 % while about 8.6 % accounts for protonated nitrogen. However, after converting GOP to GOQ, the amine percentage decreased to 15.9 % while the protonated nitrogen (quaternary ammonium salt) increased to 84.1 % indicating a successful conversion from GOP to GOQ. The deconvolution of S2p in GOT (Fig. 5l) shows two well-defined peaks at 167.8 and 169.4 eV which correspond to S-O and SO_3_, respectively [63].

**Table 3:** XPS analysis of GO and its derivatives.

Carbon	Element	Position (eV)	At (%)
GO	C1s	284.6	73.41
	O1s	532.2	26.59
GOP	C1s	284.5	71.98
	O1s	531.1	25.03
	N1s	399.8	2.99
GOQ	C1s	284.7	57.2
	O1s	530.8	40.6
	N1s	400.2	2.20
GOT	C1s	283.9	72.10
	O1s	531.1	27.10
	N1s	400.7	0.740
	S2p	168.9	1.32

### 3.6 Thermogravimetric analysis

**Fig. 6:**
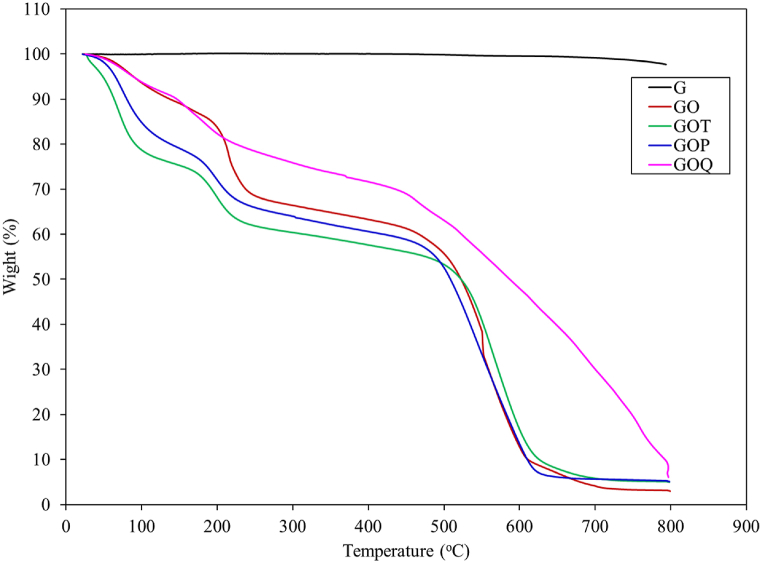
Thermogravimetric analysis of G, GO, and GO-based ion exchangers.

Thermogravimetric analysis is presented in Fig. 6. Graphite appears thermally stable with limited amounts of moisture and volatiles (3 %). The weight loss at up to 200 °C is related to the loss of moisture with 24.55, 28.4, 24.54, and 17.0 for GO, GOT, GOP, and GOQ, respectively. The weight loss between 250 and 800 °C is related to volatiles breaking away because of thermal degradation with maximum weight loss taking place around 560 °C. This is related to the breakage of covalent bonds indicating a successful covalent functionalization process. The volatile weight loss is mostly related to the emission of CO and CO_2_ from GO [12,34], and the breakage of amide moieties from GOT, GOP, and GOQ. The weight loss related to volatiles from GO, GOT, GOP, and GOQ was 72.3, 66.6, 70.3, and 73.0 %, respectively, due to volatile losses.

### 3.7 Surface acidity, basicity, and ion exchange capacity

As presented in Table 4, Boehm titrations show that GO is acidic with a –COOH content of 2.94 meq/g with the pH_zpc_ acidic (3.82), and a high CEC value (2.78 meq/g). GOT possesses an acidic surface with pH_zpc_ of 4.15 and a content of both sulfonic and unfunctionalized carboxylic accounting for a content of 2.90 meq/g, with CEC was 2.37 meq/g. The slight decrease in CEC for GOT can be related to the large space occupied by the immobilized taurine in place of the –COOH group prohibiting more uptake of Ba^2+^ ions and releasing protons into the solution. GOP, shows a basic surface with alkaline pH_zpc_ of 7.83, less content of carboxylic groups (0.39 meq/g) indicating a successful conversion of 86.7 % of carboxylic groups to amide groups. GOQ is almost neutral with pH_zpc_ of 7.01 with almost no variation in unfunctionalized carboxylic content. Both GOP and GOQ have low CEC compared with GO. GOP shows a high value of surface basicity of 10.89 meq/g which was decreased to 1.82 for GOQ indicating that about 83.3 % of the amine groups were successfully converted to quaternary ammonium salt. Finally, AEC shows a much higher value for GOQ than GO, GOP, and GOT.

**Table 4:** Surface chemical characterization of GO and its derivatives.

Carbon	pH_zpc_	CEC (mmol/g)	AEC (mmol/g)	Surface Basicity (mmol/g)	Surface functionality		
					Carboxylic (mmol/g)	Lactone (mmol/g)	Phenol (mmol/g)
G	6.70	0.050	0.04	0.56	0.33	0.160	0.004
GO	3.82	1.390	0.21	0.34	2.94	0.365	2.20
GOT	4.15	1.185	0.24	0.34	2.90^a^	0.335	2.15
GOP	7.83	0.295	0.55	10.89	0.39	0.39	2.15
GOQ	7.01	0.125	8.34	1.82	0.38	0.38	1.91

a COOH+SO_3_H.

### 3.8 Salt removal onto GO-based ion exchangers

**Fig. 7:**
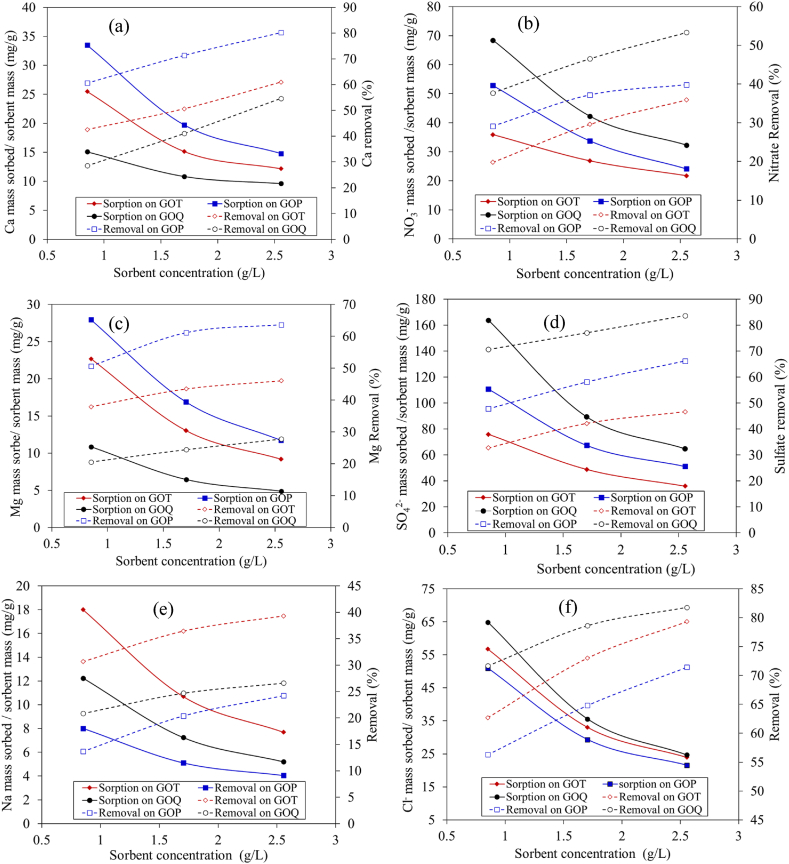
Kroeker adsorption isotherms and removal percentage of calcium nitrate [(a) Ca, (b) nitrate], magnesium sulfate [(c) Mg, (b) sulfate], and sodium chloride [(a) Na, (b) Chloride]. Initial Concentration of each metal 50 mg/L, NO_3_^−^ 154.6 mg/L, SO_4_^2−^ 197.5 mg/L, and Cl^−^ 77.1 mg/L, initial pH 7.0.

Three individual salt solutions of Ca(NO_3_)_2_ with Ca^2+^ 50 mg/L and corresponding NO_3_^−^ 155 mg/L, MgSO_4_ with Mg^2+^ 50 mg/L and corresponding SO_4_^2−^ 197.7 mg/L, and NaCl with Na^+^ 50 mg/L and corresponding Cl^−^ 77.1 mg/L were tested for Kroeker adsorption isotherm and removal (%) using GOT, GOP, and GOQ. As presented in Fig. 7(a–f), the percentage of cations and anions removal increases as sorbent mass increases due to the increase in ion exchange sites provided with the increased mass. However, the uptake of cations and anions (mg/g) decreases with the increased sorbent mass, Fig. 7a-f. Similar observations were reported for the adsorption of Cd(II), Pb(II), As(V) and Cr(VI) on a silty clay using different masses of sorbent [64]. At constant volume and concentration of solute, the amount sorbed in mg of sorbate per g of sorbent showed a decrease as the sorbent concentration increased following the Kroeker empirical isotherm, equation (2) [65].

(Eq. 2) $$m_{i}\;/m_{s}=\left(m_{oi}\;/m\right)\;\left(1-e^{-km}\right)$$

*m*_*i*_ refers to the mass of ion sorbed (mg), *m*_*s*_ is the mass of sorbent used (g), m_oi_ is the initial mass concentration of ion sorbed (mg/L), *m* is the sorbent mass concentration (g/L), and *k* is Kroeker's empirical constant (L/g). The constant *k* can be expressed as in equation (3).

(Eq. 3) $$k=-\ln\left[1-\left(m_{i}/m_{s}\right)\left(m/m_{oi}\right)\right]/\;m$$

The Kroeker empirical constant was calculated and presented in Appendix (A3). The relative standard deviations (RSD) for the empirical constant, *k*, for metal sorption ranges between 14.5 % and 43.1 % while for anion sorption, *k* values show RSD values between 14.9 % and 33.3 % indicating that the Kroeker constant K depends strongly on the initial concentration of the adsorbent [65].

The decrease in ion uptake (mg/g) with increased sorbent mass, Fig. 7(a–f) can be explained as follows. Using a small amount of sorbent, the ions are relatively in a high concentration, which competes for limited ion exchange sites on the sorbent surface, hence, attaining a high uptake. However, using a larger amount of sorbent, more ion exchange sites are provided which are not fully occupied by the ions at equilibrium leading to a decrease in ion uptake (mg/g) [66].

For the uptake of Ca^2+^ and Mg^2+^ from their respective solutions, metal sorption follows the order of GOP > GOT > GOQ while the uptake of their respective anions of NO^3−^ and SO_4_^2−^ follows the order of GOQ > GOP > GOT. GOP sorbs Ca^2+^ and Mg^2+^ via chelation while GOT sorbs them via ion exchange onto the sulfonic and unfunctionalized carboxylic groups. This was noticed from the equilibrium pH of the solution as it showed no variation for GOP but showed a decrease for GOT. The anions were adsorbed onto both GOP and GOT via an ion-pairing mechanism [18]. GOQ, on the other side, shows the best performance for the removal of both NO_3_^−^ and SO_4_^2−^ via ion exchange mechanism onto the positively charged nitrogen sites on GOQ. An extent of cations is still sorbed onto GOQ via ion pairing [66].

For the sorption of Na^+^ and Cl^−^, the sorption of Na^+^ follows the order of GOT > GOQ > GOP. Na^+^ is sorbed onto the strong cation exchanger (GOT) as it possesses negatively charged sites of –SO3^-^ on its surface, while the amount of Cl^−^ sorbed in this case is related to ion pairing [66]. GOP shows low sorption of Na^+^ and Cl^−^ because Na chelation of the amine groups of GOP is very weak [67] and the amount of Cl^−^ sorbed by ion pairing is consequently low. The anion exchanger GOQ adsorb Cl^−^ via ion exchange onto the positively charged sites of GOQ while Na^+^ is sorbed via ion pairing similarly to Mg^2+^ and Ca^2+^ as mentioned above [66]. Further investigation in terms of the effects of pH, contact time, and concentration on the removal of salts from both synthetic solutions and brackish water will be investigated shortly. Starting with graphite, initial cost analysis shows an estimate of 698, 532, and 786 €/kg of GOT, GOP, and GOQ, respectively. This cost does not include chemical reuse. However, an industrial cost analysis will be carried out shortly. Such costs are comparable to commercially available ion exchangers, however, with the advantage for GO-ion exchangers of being robust at higher values of both temperature and pH, unlike polymer-based and silica-based ion exchangers [12].

## 4 Conclusion

GO was produced from graphite using an oxidative exfoliation method. GO was successfully functionalized to produce a strong cation exchanger (GOT), and a chelating cation exchanger (GOP) using taurine and PEHA. The amine groups on GOP were successfully converted to quaternary ammonium salt (GOQ). TGA confirmed the covalent functionalization of the ion exchangers produced, and robustness towards high temperature. Nitrogen is mostly found as amine groups on GOP but as quaternary ammonium salt on GOQ. For the experimental conditions for desalination, in this study, GOP shows the best performance of the removal of Ca^2+^ (80.2 %) and Mg^2+^ (64 %) while GOT shows the best removal of Na^+^ (30 %). For anions, GOQ shows the best removal of NO_3_^−^ (53.5 %), SO_4_^2−^ (84 %), and Cl^−^ (81.8 %). The ion exchangers produced, in this study, seem promising for desalination with the advantage of being robust compared with the polymeric ion exchange resins and silica-based ion exchangers.

## CRediT authorship contribution statement

**Mahmoud A. Al Azwani:** Writing – original draft, Investigation, Data curation. **Saleh Al Busafi:** Supervision, Methodology, Conceptualization. **El-Said I. El-Shafey:** Writing – review & editing, Supervision, Methodology, Conceptualization.

## Data availability

Data included in article/supp. Material/referenced in the article.

## Declaration of competing interest

The authors declare that they have no known competing financial interests or personal relationships that could have appeared to influence the work reported in this paper.
