# Supplementary figures and images for: Preparation and characterization of graphene oxide-based cation, chelating, and anion exchangers for salt removal
Source: Heliyon. 2025 Jan 17;11(3):e42070. doi: 10.1016/j.heliyon.2025.e42070 (PMC11808519; doi:10.1016/j.heliyon.2025.e42070)

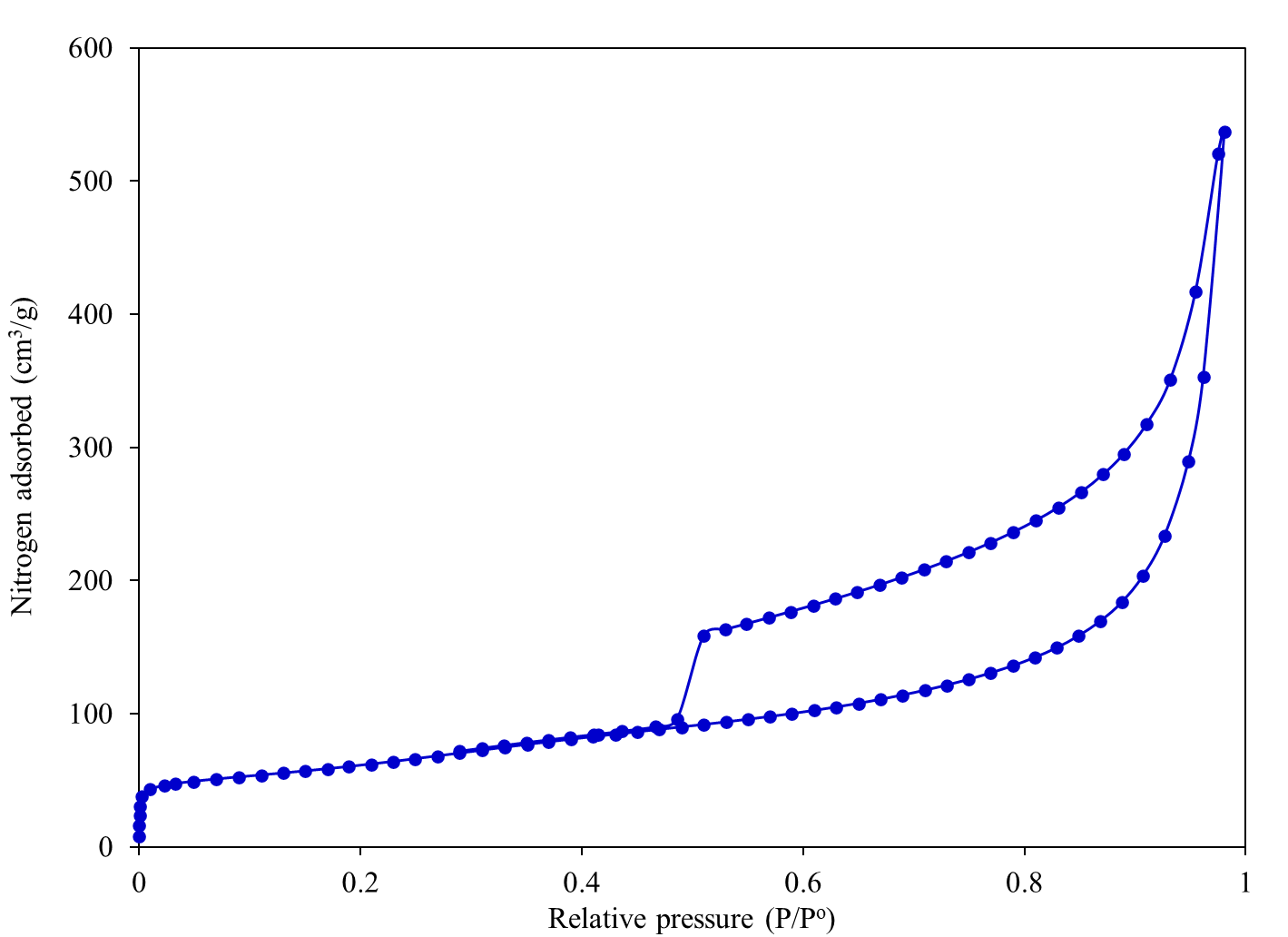


(A)


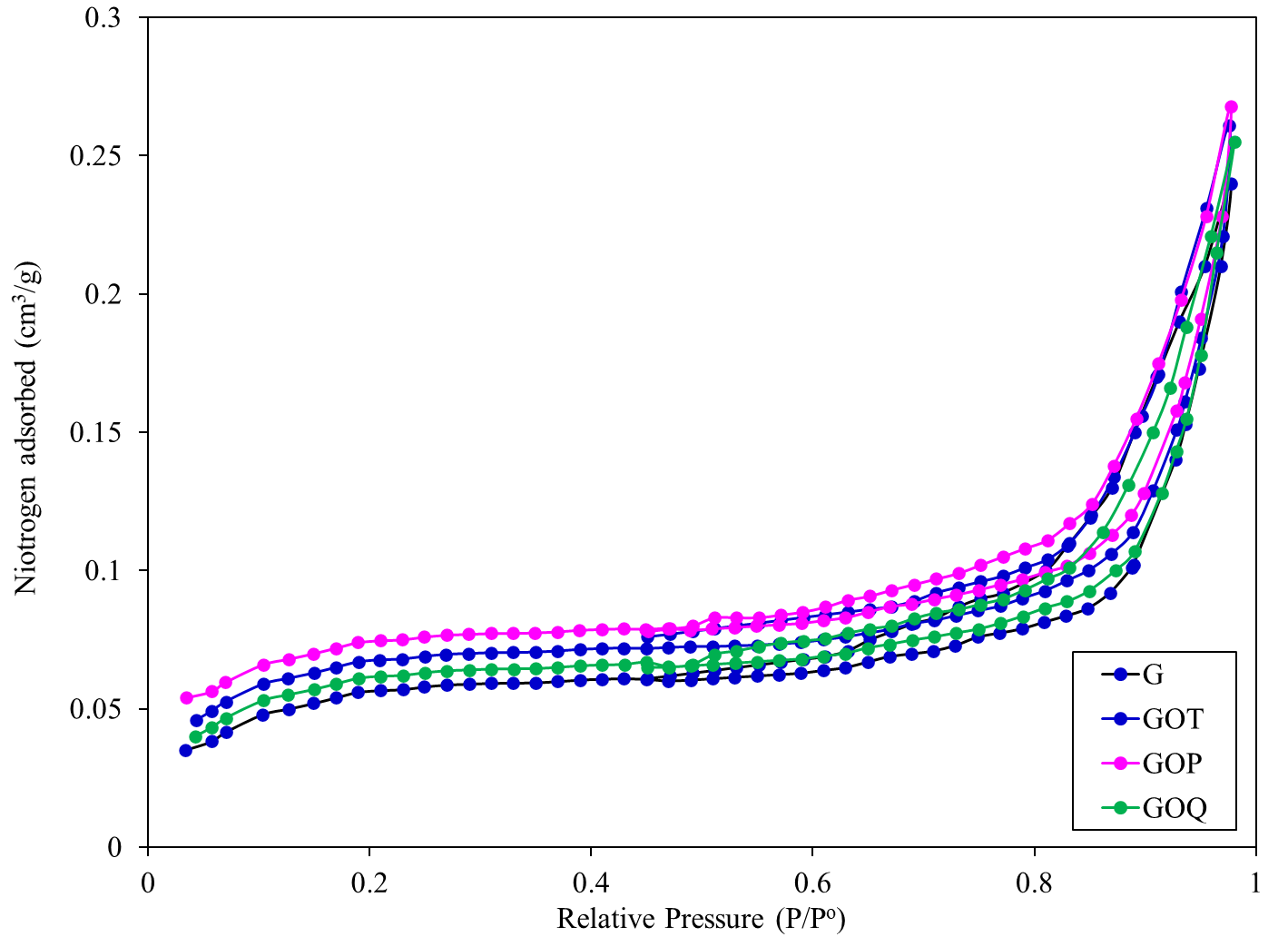


(B)

Figure A1. Nitrogen adsorption isotherms at 77 K on (A) GO, and (B) graphite and GO-based ion exchangers

Supplement: Multimedia component 1 [file mmc1.docx]

(a)


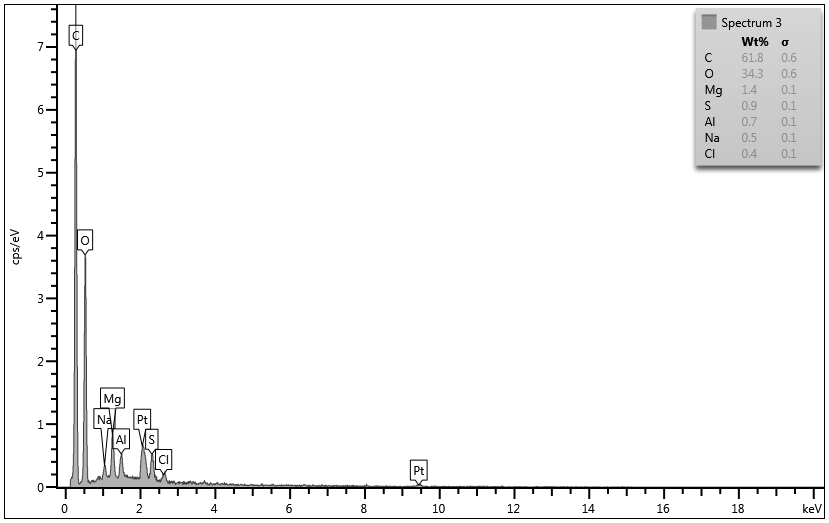


(b)


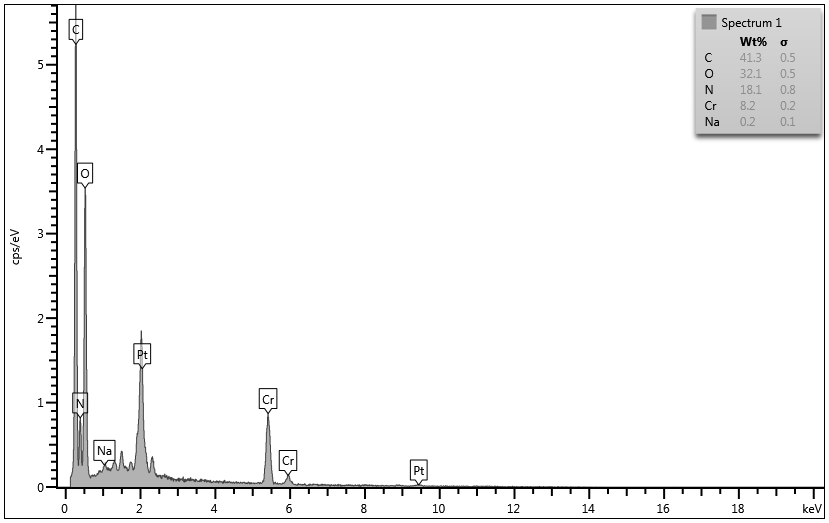


(c)


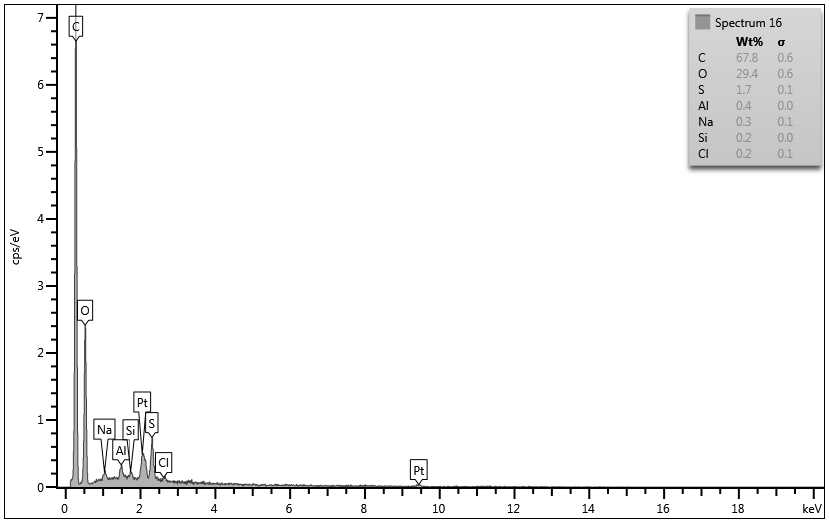


Figure A2. EDS spectra of (a) GO, (b) GOP, and (c) GOT.

Supplement: Multimedia component 2 [file mmc2.docx]
