# Supplementary material for: Preparation and characterization of graphene oxide-based cation, chelating, and anion exchangers for salt removal
Source: Heliyon. 2025 Jan 17;11(3):e42070. doi: 10.1016/j.heliyon.2025.e42070 (PMC11808519; doi:10.1016/j.heliyon.2025.e42070)
Supplement: Multimedia component 3 [file mmc3.docx]

| Sorbent | Sorbate | K (L/g) | Sorbate | K (L/g) |
| --- | --- | --- | --- | --- |
| GOT | Ca | 0.48 ± 0.15 | NO_3_^-^ | 0.0022 ± 0.0010 |
|  | Mg | 0.38 ± 0.16 | SO_4_^2-^ | 0.0027 ± 0.0006 |
|  | Na | 0.0041 ± 0.0008 | Cl^-^ | 0.017 ± 0.0038 |
| GOP | Ca | 0.70 ± 0.21 | NO_3_^-^ | 0.0022 ± 0.0006 |
|  | Mg | 0.51 ± 0.16 | SO_4_^2-^ | 0.0044 ± 0.0011 |
|  | Na | 0.0019 ± 0.0007 | Cl^-^ | 0.0135 ± 0.0027 |
| GOQ | Ca | 0.34 ± 0.05 | NO_3_^-^ | 0.004 ± 0.0010 |
|  | Mg | 0.19 ± 0.07 | SO_4_^2-^ | 0.0076 ± 0.0015 |
|  | Na | 0.0024 ± 0.0004 | Cl^-^ | 0.0195 ± 0.0029 |

Table A3. Kroeker empirical constant values
